# Retraction: Natural gas price prediction based on artificial intelligence models

**DOI:** 10.1371/journal.pone.0343466

**Published:** 2026-02-23

**Authors:** The PLOS One Editors

After this article [1] was published, the following concerns were noted:

- The articles cited as References 3, 4, 5, 7, 8, 9, 10, 16, 17, 18, 20, 29, 32, 34, 49, 55, 57, 60, 61, 73, and 74 do not appear to support the corresponding cited statements.
- The article cited as Reference 3 was retracted before [1] was published.
- Potential non-compliance with the PLOS policy on Artificial Intelligence Tools and Technologies.

The first author acknowledged that errors occurred in verifying references during manuscript preparation and that the authors failed to declare the use of artificial intelligence tools for language polishing and text sorting.

The *PLOS One* Editors retract this article due to the cumulative issues which raise concerns about its reliability and provenance.

XL and MS agreed with the retraction. MT, YF, TF, and ZW either did not respond directly or could not be reached.
